# Fluoride Levels in Supply Water from the Canary Islands Region

**DOI:** 10.3390/foods12040745

**Published:** 2023-02-08

**Authors:** Inés A. Revelo-Mejía, Samuel Alejandro-Vega, Soraya Paz-Montelongo, Daniel Niebla-Canelo, Santiago Cerdán-Pérez, Carmen Rubio-Armendáriz, Ángel J. Gutiérrez-Fernández, Arturo Hardisson, Rubí Rodríguez-Díaz, Cintia Hernández-Sánchez

**Affiliations:** 1 Faculty of Odontology, Universidad Antonio Nariño, 37-94 Calle 58 A Bis, Bogota 111321, Cauca, Colombia; 2 Area of Toxicology, Universidad de La Laguna, 38071 La Laguna, Tenerife, Canary Islands, Spain; 3 Grupo Interuniversitario de Toxicología Alimentaria y Ambiental, Universidad de La Laguna, 38071 La Laguna, Tenerife, Canary Islands, Spain; 4 Human Reproduction Unit, Hospital Universitario de Canarias, Carretera Ofra S/N, 38320 La Laguna, Tenerife, Canary Islands, Spain; 5 Area of Obstetrics and Gynecology, Universidad de La Laguna, 38071 La Laguna, Tenerife, Canary Islands, Spain; 6 Area of Preventive Medicine and Public Health, Universidad de La Laguna, 38071 La Laguna, Tenerife, Canary Islands, Spain

**Keywords:** Canary Islands, fluoride, supply water, risk assessment

## Abstract

The Canary Islands, located in the Atlantic Ocean, are an archipelago of volcanic origin which, for decades, has been affected by natural fluoride contamination in the water supply of some of its islands, mainly the island of Tenerife. In addition, recent volcanic eruptions in the archipelago and the increased demand for water supply have led to an increase in the fluoride content in other areas which, historically, were not affected. Fluoride content was determined in 274 water supply samples from the most populated islands of the Canary Islands (Tenerife and Gran Canaria) collected during the months of June 2021 to May 2022. The samples were analysed by fluoride ion selective potentiometry. The highest concentrations in Tenerife were found in the municipalities of Sauzal (7.00 mg/L) and Tegueste (5.39 mg/L), both water samples are over the parametric value of 1.5 mg/L set in the supply water legislation. In the Gran Canaria Island, the highest fluoride levels were found in Valsequillo and Mogán with 1.44 mg/L in both locations, but under the parametric fluoride value abovementioned. Consumption of just 1 L of water per day in the El Sauzal area would result in a contribution rate of 77% for adults and children over 15 years of age (Upper Level value of 7 mg/day) and 108% for children 9–14 years of age (UL value of 5 mg/day). The contribution rates increase considerably, reaching or exceeding 100% of the reference value (UL) with increasing consumption of 1 to 2 L of water per day. Therefore, it is considered that there is a health risk of overexposure to fluoride on the island of Tenerife. In the case of the island of Gran Canaria, it has been shown that even the consumption of 2 litres of water per day does not confer contribution rates that pose a health risk.

## 1. Introduction

Fluorine is an element that belongs to the halogen group and is the most reactive element in the periodic table. The high reactivity of this element allows it to react with a large number of compounds and elements, producing fluorinated derivatives such as hydrogen fluoride and calcium fluoride [1,2]. In the past, this element was considered a trace element, a classification that is now changing. The fluoride content in the organism ranges from 2.6 to 4 g and is mainly found in bone tissue, due to its high affinity with calcium, viscera, thyroid, plasma, etc [3].

This element is widely distributed in nature, being found in minerals and rocks of volcanic origin. It is precisely volcanic activity that is responsible for the largest emissions of fluoride into the environment, either through fluoride-containing gases or through leaching and erosion of fluoride-containing minerals. However, human activity can also increase the fluoride content in the environment through the use of phosphate fertilisers, industrial activity, water fluoridation, toothpaste and mouthwash residues, and the use of polyvinyl fluoride-based kitchen utensils [1,2,4,5,6].

Fluoride is mainly absorbed orally through the consumption of water, other beverages and some fluoride-rich foods. The digestive route is the main route of fluoride absorption. The mechanism of absorption is by simple diffusion, with about 75–80% of available fluoride being absorbed in the small intestine and the remainder in the stomach. It should be noted that water, being in a liquid state and in the absence of other elements, favours the absorption of the fluoride it contains, being 95–97%. Meanwhile, the absorption of fluoride from solid foods is lower, being 60–70% [7,8,9].

The fluoride ion has been referenced in multiple studies for its beneficial health effects such as strengthening the mineral structure of teeth and tooth enamel, inhibiting cariogenic bacteria and reducing osteoporosis by increasing bone structure [3,10,11,12,13]. However, although these beneficial effects are well known, there are multiple toxic effects of this anion, which can cause amino acid disorders due to the combination of fluoride with phenylalanine or tyrosine, a powerful complexing agent of essential elements such as Ca or iodine, reduced IQ in children, damage to reproduction and the more well-known effects such as dental fluorosis and skeletal fluorosis [8,9,14,15,16,17,18,19,20].

Despite its toxic effects, this ion remains uncontrolled in several regions and the consequences for the health of the population can be serious. The case of regional fluorosis affecting the water supply of the Canary Islands, an outermost region in the Atlantic Ocean belonging to Spain, has been known for decades. This problem is due to the volcanic origin of the islands and to the use of water from galleries [4,6,21,22]. However, it affects some islands more than others and although in recent years actions have been taken to reduce the fluoride content in the water supply of the islands, the problem is still persistent in some areas such as, for example, the north of the island of Tenerife. For this reason, this study has been carried out with the aim of (1) determining the fluoride content in the water supply of the island of Tenerife and the island of Gran Canaria and (2) evaluating the intake of fluoride from the consumption of this water by the population of the islands.

## 2. Material and Methods

### 2.1. Samples and Sampling Areas

**Figure 1:**
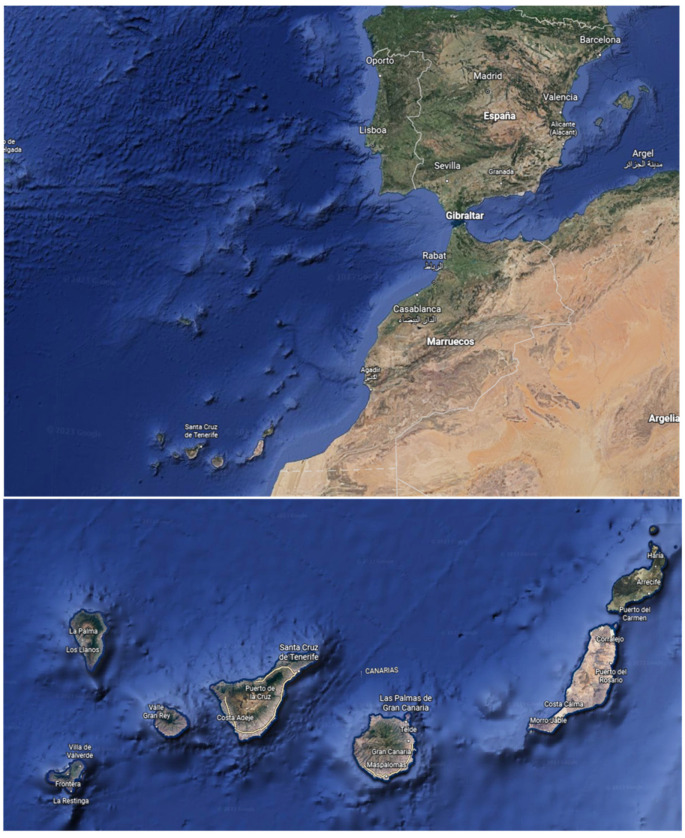
Canary Islands situation (obtained from Google Earth™ software (version 9.175, Google, Mountain View, CA, USA)).

A total of 274 samples were collected in Gran Canaria and Tenerife islands from Canary Islands archipelago, 78 of those were taken in the former and 196 in the latter one (Table 1) (Figure 1). Three replicates were taken from each sampling point. Gran Canaria’s sampling locations were divided in four areas based on demography and topography, meanwhile only three zones were created in Tenerife. The Table 2 summarized the water sources of the collected samples.

The island of Tenerife (28°16′07″ N 16°36′20″ W), is the largest and most populated of the Canary archipelago. The drinking water supply of the island of Tenerife is based on rainwater infiltrations that pass through the island’s generally porous volcanic soil and accumulate in underground galleries. The island of Gran Canaria (27°57′31″ N 15°35′33″ W), is the second most populated island of the Canary Islands. According to the Hydrological Plan of Gran Canaria, around 50% of the island’s water resources come from seawater desalination, while water extracted from underground galleries only represents 35% and a 15% from surface water sources [23].

The Canary Islands are an archipelago with a volcanic origin. Volcanism is one of the most important factors in the occurrence of fluorides in water [24,25]. This study has chosen two of the volcanic islands where the presence of fluorides in their water has previously been found, especially in Tenerife [26,27,28,29]. It is indisputable that population is linked to water demand, so a high inhabitants density generates overexploitation of its water resources. In relation to island aquifers, when natural recharge from rainfall is lower than reserves, this could lead to a significant increase in fluoride levels [30]. 

In addition, the recent eruptions In the Canary Islands (submarine eruption of the Tagoro Volcano on El Hierro in October 2011, terrestrial eruption of the Tajogaite Volcano on La Palma in September 2021) together with the unified model of the origin of the Canary Islands proposed by Anguita and Hernan [31], highlights the importance of adequate monitoring and control of the island’ water supply.

**Figure 2:**
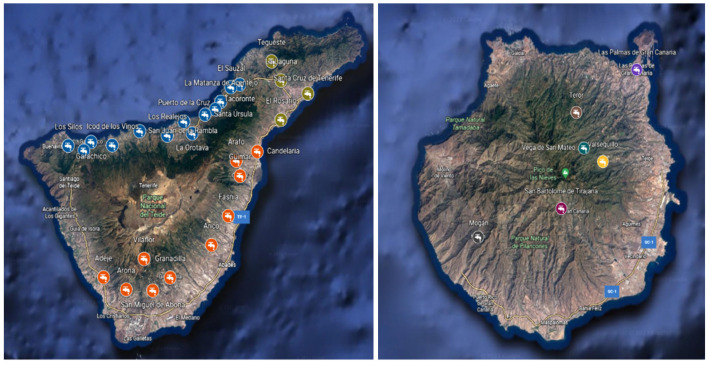
Sampling locations in Tenerife and Gran Canaria islands (obtained from Google Earth™ software).

The sampling locations are showed in the Figure 2. The samples consist of tap water collected from different businesses and private homes by allowing the water to flow for 5 min prior to collection. In addition, these samples were collected in airtight plastic containers, to avoid the loss of fluoride by ionic diffusion in silicates. These containers were kept cold and the analysis were performed within a period not exceeding 5 days after collection.

### 2.2. Analytical Method

In order to proceed with the analytical method orthophosphoric acid 0.75 M as conditioner solution is needed [32], it was prepared from concentrated orthophosphoric acid 85% (Honeywell-Fluka, Germany). In addition, a fluoride calibration curve (10-5 M, 10-4 M, 10-3 M, 10-2 M, 10-1 M) in conditioner solution, made from NaF analytic purity (Merck, Germany). The fluoride analysis was carried out by Ion Selective Potentiometry with an ISE potentiometer model HACH SensION-MM340 (HACH, Düsseldorf, Germany) and a F^−^ ion selective electrode (HACH ISE F-9655C, Düsseldorf, Germany). Which instrumental parameters were: range (0.01–19,000 mg/L), pH range (4–8), linear range (0.1–19,000 mg/L), slope (59 mV/pF) and operating temperature (5–50 °C). Possible interferences: Fe^3+^ and Al^3+^, removed by the conditioning solution. Samples were prepared with 5 mL of the conditioner solution and 25 mL of the sample.

### 2.3. Method Quality Control and Validation

The precision of the method was assessed under reproducibility conditions by standard addition method. Once Samples’ F^−^ concentration was determined a known amount of fluoride was added. This process was repeated 15 times a day on 3 different days.

The repeatability values in terms of relative standard deviation (RSD) were 0.50% with a reproducibility of 2.70%. The recovery rate was 99% with an RSD lower than 5%. The values were adequate, and the recovery rate was satisfactory

### 2.4. Statistical Analysis

The statistical analysis was carried out using GraphPad Prism (GraphPad, San Diego, CA, USA) version 8.0.1. The distribution of the results was studied by the application of the normality and lognormality tests (Anderson-Darling, D’Agostino and Pearson, Shapiro-Wilk and Kolmogorov-Smirnov tests). The results reported that the data follow a non-normal distribution [32]. Once the non-normality of the results had been demonstrated, non-parametric tests (Mann-Whitney and Kolmogorov-Smirnov tests) were applied. 

The statistical study was carried out with the aim of studying significant differences (*p* < 0.05) in fluoride content between municipalities, areas (metropolitan, north, south and midlands) and between islands (Tenerife vs. Gran Canaria).

### 2.5. Dietary Intake Assessment

The assessment of dietary intake has been based on obtaining the estimated daily intake (EDI) (Equation (1)) of fluoride from the consumption of three different amounts of water supply (1, 1.5 and 2 L/day) by the adult population.

(1) $$\mathrm{EDI}\;\left(\frac{\mathrm{mg}}{\mathrm{day}}\right)=\mathrm{Fluoride}\;\mathrm{concentration}\;\left(\frac{\mathrm{mg}}{L}\right)\times \mathrm{Water}\;\mathrm{consumption}\;\left(\frac{L}{\mathrm{day}}\right)$$

Once the EDI is obtained for each consumption scenario considered and for each area analysed, the percentage contribution (%) is obtained (Equation (2)) considering for them the UL (tolerable upper intake level) values established for fluoride by the EFSA (European Food Safety Authority).

(2) $$\mathrm{Contribution}\;\left(\%\right)=\left(\mathrm{EDI}\;\left(\frac{\mathrm{mg}}{\mathrm{day}}\right)/\mathrm{UL}\left(\frac{\mathrm{mg}}{\mathrm{day}}\right)\right)\times 100$$

The UL is defined as the maximum tolerable intake amount from chronic consumption that does not pose a risk or adverse effect to human health. The UL value for children (9–14 years) is 5 mg/day, and the value for children (15–17 years) and adults is 7 mg/day.

## 3. Results and Discussion

### 3.1. Fluoride Content in Tenerife Island

**Figure 3:**
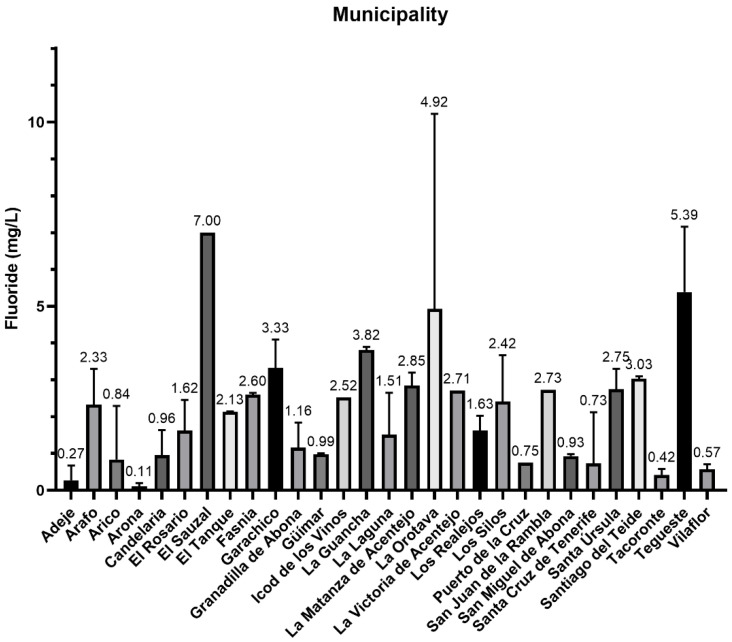
Fluoride content (mg/L) by municipalities in the Tenerife’s island.

**Figure 4:**
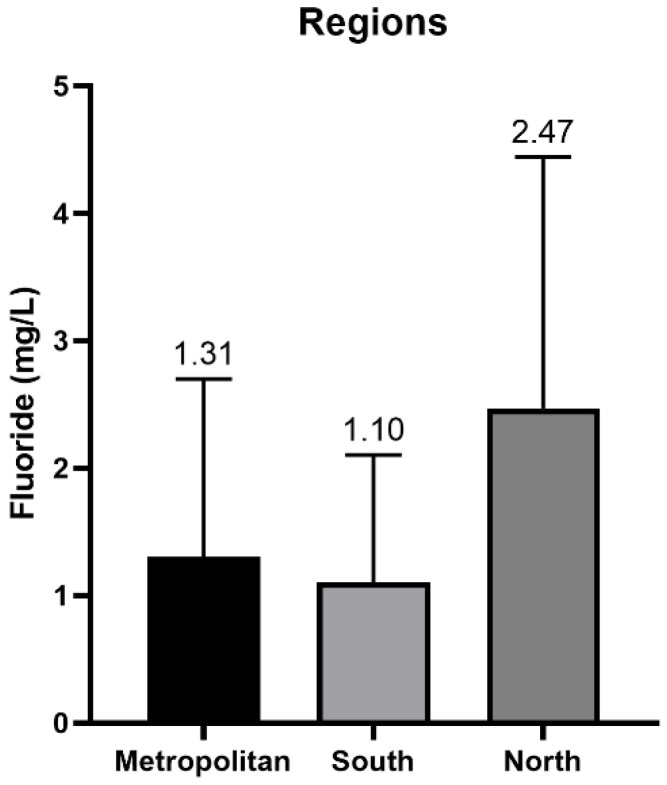
Fluoride content (mg/L) by regions in the Tenerife’s island.

Table 3 shows the fluoride concentration determined in different municipalities of the island of Tenerife. Figure 3 shows the comparison in fluoride content between the different municipalities. Figure 4 shows the mean concentrations and comparison in fluoride content between the three areas of the island of Tenerife (north, south, metropolitan).

It should be noted that on the island of Tenerife, many municipalities exceed the parametric level (1.5 mg/L) established in the aforementioned regulation (98/83/CE Directive of the European Commission) [33]. The percentage of municipalities exceeding this parametric level is 65.5%. These data confirm the problem of endemic fluorosis on the island of Tenerife. As for the municipalities with the highest average concentrations, El Sauzal is the municipality with the highest average fluoride concentration (7 mg/L), followed by Tegueste (5.39 mg/L) and La Orotava (4.92 mg/L). The water from El Sauzal and La Orotava comes from galleries, so it is groundwater. However, the water collected in Tegueste comes from a mixture between galleries and dug well. In contrast, the areas with the lowest concentrations are Arona (0.11 mg/L), Adeje (0.27 mg/L) and Tacoronte (0.42 mg/L). In the case of the Arona and Adeje areas, both use water from desalination plants, from dug wells with little casing, and even from gallery water. In the case of Tacoronte, the water in the collection area comes from a dug well.

The statistical analysis confirmed the existence of significant differences in fluoride content between the northern and southern areas (*p* < 0.0001), as well as between the northern and metropolitan areas (*p* < 0.0001). However, no significant differences were detected in the fluoride content in the metropolitan area vs. the southern area (*p* = 0.4271), which may be due to the fact that in the municipality of Santa Cruz part of the water supply comes from desalination plants, as is the case in many municipalities in the southern area of Tenerife.

It is noteworthy that the northern regions of the island continue to have the highest fluoride concentrations (Figure 4), a problem that has persisted for many decades. Thus, the study carried out by Rubio et al. in 2020 recorded concentrations of 4.59, 2.50 and 5.55 mg/L in Icod de los Vinos, San Juan de la Rambla and La Guancha, respectively [27]. Hardisson et al. in 2001 found concentrations of 4.59, 2.50 and 5.55 mg/L in the same locations, and also presented a history of results, where the fluoride concentration of the Vergara gallery from 1976 to 1997 is highlighted, where no result is lower than 6 mg/L [26]. This is of great importance, since historically this has been the main source of drinking water supply in the northern part of the island and is the reason why this region has a high incidence of fluorosis, such is the situation that in 2021 restrictions had to be established on the consumption of drinking water in the municipality of La Guancha [34].

The presence of volcanic minerals in the main water galleries supplying the island of Tenerife continues to be a notorious source of fluorides. Although, as can be seen in Figure 4, municipalities in the south that are supplied, in some areas, with water from desalination plants, have lower concentrations of this ion. For this reason, the installation of water treatment plants is a viable option to considerably reduce the fluoride content in the water supply of the island of Tenerife. However, the high energy costs of the desalination process mean that more sustainable methods of supplying the water demand of the population are being sought, especially in isolated island systems such as the Canary Islands [35]. Undoubtedly, the most sustainable method is to make use of the available water from previously excavated galleries, including the Barranco de Vergara gallery in the municipality of La Guancha, Tenerife, the largest in the Canary Islands but also the one with the highest concentration of fluoride [26,36]. This is why the waters of the municipality of La Guancha are historically the ones with the highest concentration of fluorides [26,27] where there are still currently water supply areas in this municipality with restricted water consumption due to high levels of fluoride [37]. 

### 3.2. Fluoride Content on the Island of Gran Canaria

Table 4 shows the mean fluoride concentrations and standard deviations in water samples from the island of Gran Canaria divided by metropolitan areas, south, midlands and north. Half of Gran Canaria’s water consumption is desalinated water, this kind of resource is mainly used at the municipalities of San Bartolomé de Tirajana, Las Palmas de Gran Canaria and other Metropolitan town halls [23]. Therefore, the concentration of fluoride in these areas is expected to be lower than the areas with a water supply based mainly on ground water (Table 3).

As dictated by the 98/83/CE Directive of the European Commission the parametric value of fluoride in water intended for human consumption is 1.5 mg/L. Therefore, the fluoride’s concentration registered in each municipality was compared with this parametric value [33].

**Figure 5:**
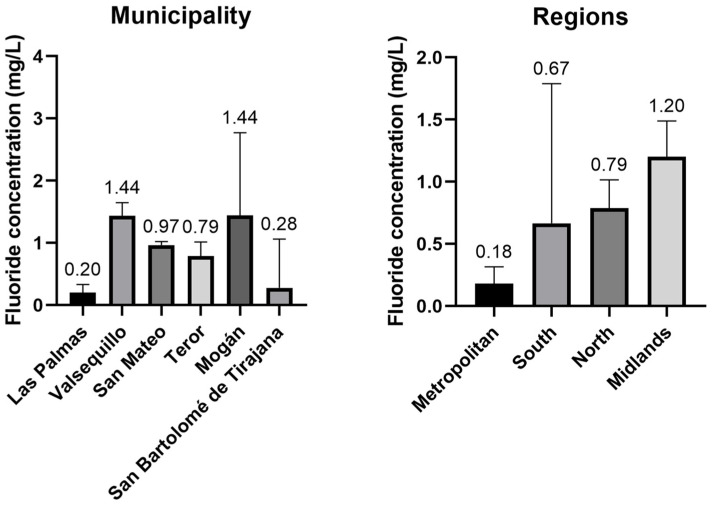
Comparison of the fluoride content (mg/L) between the municipalities and the regions of the Gran Canaria’s island.

Even though the mean concentration in all the Gran Canaria’s studied town halls was under this parametric value, 8% of the 78 samples exceed the 1.5 mg/L concentration (Figure 5). These samples were gathered only in three municipalities as 50% of Mogán’s samples had more than 1.5 mg/L of fluoride, meanwhile 20% of Valsequillo’s samples and 5% of San Bartolome de Tirajana’s had a value bigger than the parametric level.

The Mann-Whitney’s tests carried out by the studied municipalities show that there are significative differences (*p* < 0.05) between all town halls except Mogán-Vega de San Mateo, Mogán-Teror, Mogan-Valsequillo and Vega de San Mateo-Teror pairs. Mogán’s public water supply does not have any influx of desalinated water and most of it come from underground water collected in the higher altitudes of the island next to Midlands’ municipalities that could be the reason behind the absence of difference in those pairs [23].

Meanwhile the same tests carried out by Gran Canaria’s areas show that there are significative differences (*p* < 0.05) between all studied zones except the Metropolitan-South pair. This was expected as both zones are the ones that have a big percentage of their water consumption supplied by desalinated water instead of underground one, as it is needed to satisfy the high demand of the most populated and touristic centre parts of the island [23].

### 3.3. Comparison of Fluoride Content in Tenerife and Gran Canaria

**Figure 6:**
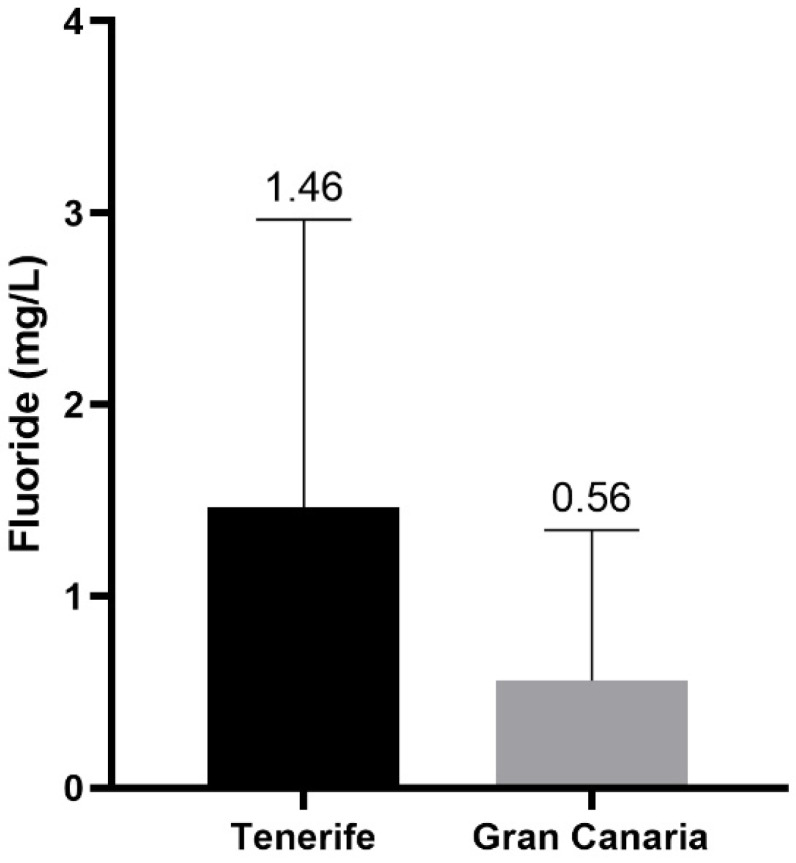
Fluoride content (mg/L) in the Tenerife and Gran Canaria’s islands.

Figure 6 shows the comparison of fluoride content between the island of Tenerife and the island of Gran Canaria. As can be seen, the island of Tenerife has the highest mean fluoride concentration (1.46 mg/L) while the island of Gran Canaria has a concentration of 0.56 mg/L, meeting the parametric value of fluoride indicated above. Furthermore, the statistical study confirmed the existence of significant differences in fluoride content between the two islands (*p* < 0.0001). These differences are due to the fact that on the island of Tenerife, most of the water supply comes from underground galleries which, as mentioned above, contain volcanic minerals rich in fluoride, which increases the content of this ion in the water. Meanwhile, the island of Gran Canaria, with fewer water resources, has opted to use water from desalination plants.

This comparison shows that, as far as fluoride content is concerned, the use of desalination plants is a valuable method for the drastic reduction in this ion in the water supply. 

Although both cases involve volcanic islands, the effects on groundwater bodies are different. On the one hand, according to the Gran Canaria Hydrological Plan, the main effects on groundwater bodies are the areas affected by nitrates of agricultural origin [38]. The Gran Canaria Hydrological Plan also highlights the decrease in the island’s water resources, with up to 50% of the island’s current water demand being supplied by desalination [23]. 

On the other hand, the Tenerife Hydrological Plan establishes that the main effects on groundwater are: areas affected by nitrate pollution of agricultural origin [38], areas with a greater risk of being affected by seawater intrusion processes and areas affected by remaining volcanic activity. The latter are those that produce contamination by natural processes of volcanic origin in which drinking water may be affected, as in the case of fluorides. The desalination of seawater in the hydraulic balance of the island, based on the Tenerife Hydrological Plan, is only 14% [39], compared to 81% of underground origin.

Supplying desalinated water directly from the sea in an island system reduces the risk of exposure to fluoride in drinking water, while at the same time increasing the price of water [35]. 

A study carried out by Kairala et al. [40] in Nepal shows fluoride concentrations below 0.50 mg/L, with some samples slightly exceeding this value. Another study in Bangladesh, using groundwater samples, shows concentrations ranging from 0.01 mg/L to 15 mg/L (wet season) and from 0.01 to 16.11 mg/L (dry season) [41]. In their literature review, Lacson et al. [42] compile data on fluoride concentrations in different regions of the world. The most affected regions are African, Middle-East-to-South-East Asian, and American belts. 

The concentrations reported in this study show varying concentrations, as do the authors cited. Therefore, comparing the results obtained in this study with similar regions is complex, as fluoride content can be affected by multiple factors, both natural and anthropogenic. As for example, studies carried out in Iran show that the large variability in fluoride concentration in water is due to multiple physico-chemical factors such as pH, turbidity, presence of certain anions and cations, sodium content, etc. [43,44]. However, what is demonstrated is that the geology of the region, characterised by being a volcanic region, causes a higher concentration of this ion. 

### 3.4. Dietary Intake Assessment

Table 5 shows the evaluation of fluoride intake in children (9–14 years and 15–17 years) and adults on the island of Tenerife. The consumption of 1 L of water per day in the municipality of El Sauzal already confers 100% of the reference value (UL, upper intake level) established by EFSA at 7 mg/day, which means that, following the recommendations of 2 L/day of water consumption, the adult inhabitants of El Sauzal (northern region) would be at risk of high exposure to fluoride. The same is true for water consumption in the area of Tegueste (metropolitan region), who with 1.5 L/day would be exposed to more than 100% of the UL for adults. The scenario of consumption of 2 L of water per day would mean that the UL would be exceeded in 4 municipalities of the island of Tenerife for the child population aged 15–17 years and the adult population.

For children aged 9 to 14 years, whose UL is 5 mg/day, the consumption of 1 L of water per day in the areas of El Sauzal and Tegueste would mean exceeding the established UL, while, by increasing consumption to 1.5 litres per day, the UL value for the population aged 9 to 14 years is greatly exceeded in a total of 4 municipalities and, in the case of consumption of 2 L/day, this would amount to a total of 12 municipalities in which the UL for this population group would be greatly exceeded.

Considering the harmful effects of fluoride, which go beyond the well-known dental and bone fluorosis, the consumption of bottled water by the underage population in the municipalities indicated is recommended.

On the other hand, with regard to the intake of fluoride from the consumption of water supplies on the island of Gran Canaria (Table 6), no consumption scenario, both by the adult population and children (9 to 17 years of age), exceed 100% of their respective UL. Therefore, the consumption of the water supply in the sampled areas of Gran Canaria would not pose a health risk to this population group.

## 4. Conclusions

The parametric values for fluoride in the water supply (1.5 mg/L) are exceeded in 65.5% of the municipalities of the island of Tenerife, most of them being municipalities in the north. On the island of Gran Canaria no municipality exceeds this parametric value.

As for the dietary exposure to fluoride of children (9 to 17 years old) and adults, in some municipalities of Tenerife the UL is exceeded or close to 100%. It is necessary to recommend the consumption of bottled water in some regions of the island of Tenerife, especially by the child population.

The problem of fluoride in the water supply of the island of Tenerife remains despite the knowledge of the problem and its potential harm to human health. However, it has been shown that, in those municipalities whose water supply came from desalination plants, fluoride levels were significantly lower than those municipalities whose water supply came from groundwater. In comparison with the fluoride levels recorded on the island of Gran Canaria, the effectiveness of using water from desalination plants or even mixing water with a higher fluoride content with water containing lower fluoride concentrations is demonstrated.

According to the results obtained, infants and children in the municipalities of the north of Tenerife have a higher risk of presenting health impacts induced by the consumption of water containing fluoride content compared with adults.

## Figures and Tables

**Table 1:** Areas sampled on the island of Tenerife and the island of Gran Canaria.

**Tenerife**		
**Area**	**Municipality**	**No. Samples**
South	Adeje	9
	Arafo	5
	Arico	7
	Arona	2
	Candelaria	8
	Fasnia	3
	Granadilla de Abona	13
	Güímar	2
	San Miguel de Abona	5
	Vilaflor	3
North	El Sauzal	1
	El Tanque	2
	Garachico	2
	Icod de los Vinos	1
	La Matanza de Acentejo	5
	La Orotava	3
	La Victoria de Acentejo	1
	Los Realejos	7
	Los Silos	2
	Puerto de la Cruz	1
	San Juan de la Rambla	1
	Santa Úrsula	2
	Tacoronte	6
Metropolitan	El Rosario	11
	La Laguna	49
	Santa Cruz de Tenerife	38
	Tegueste	2
**Total**		**196**
**Gran Canaria**		
**Area**	**Municipality**	**No. Samples**
Metropolitan	Las Palmas	30
South	Mogán	10
	San Bartolomé de Tirajana	20
Midlands	Valsequillo	5
	San Mateo	5
North	Teror	8
**Total**		**78**

**Table 2:** Water sources of the collected samples from Tenerife and Gran Canaria islands.

**Tenerife**	**Water Source**					
**Area**	**Desalation**	**Surface**	**Digged Well**	**Dug Well**	**Gallery**	**Spring**
Metropolitan	55	0	14	20	69	9
North	0	0	1	0	7	0
South	7	0	0	0	17	0
Total	62	0	15	20	90	8
**Gran Canaria**	**Water Source**					
**Area**	**Desalation**	**Surface**	**Digged Well**	**Dug Well**	**Gallery**	**Spring**
South	3	2	23	3	0	0
Metropolitan	20	1	2	7	1	0
Midlands	0	10	0	0	0	0
North	0	0	5	3	0	0
Total	23	13	30	11	1	0

**Table 3:** Mean concentrations, standard deviations (SD), maximum and minimum values of fluoride in water samples from Tenerife.

Area	Municipalities	Concentration F- (mg/L)			
		Mean	Min	Max	SD
Metropolitan	El Rosario	1.62	0.47	2.61	0.83
	La Laguna	1.51	0.03	6.04	1.13
	Santa Cruz de Tenerife	0.73	0.02	5.62	1.39
	Tegueste	5.39	4.14	6.64	1.77
North	El Sauzal	7.00	7.00	7.00	0
	El Tanque	2.13	2.12	2.14	0.01
	Garachico	3.32	2.78	3.87	0.78
	Icod de los Vinos	2.52	2.52	2.52	0
	La Guancha	3.82	3.75	3.91	0.08
	La Matanza de Acentejo	2.85	2.55	3.45	0.35
	La Orotava	4.92	0.65	10.86	5.30
	La Victoria de Acentejo	2.71	2.71	2.71	0
	Los Realejos	1.63	1.03	2.21	0.40
	Los Silos	2.41	1.53	3.30	1.25
	Puerto de la Cruz	0.75	0.75	0.75	0
	San Juan de la Rambla	2.73	2.73	2.73	0
	Santa Úrsula	2.75	2.36	3.14	0.55
	Tacoronte	0.42	0.30	0.74	0.16
South	Adeje	0.27	0.03	1.08	0.40
	Arafo	2.32	1.34	3.34	0.97
	Arico	0.84	0.02	3.66	1.46
	Arona	0.11	0.05	0.17	0.08
	Candelaria	0.96	0.19	1.82	0.67
	Fasnia	2.60	2.54	2.63	0.05
	Granadilla de Abona	1.16	0.04	1.89	0.68
	Güimar	0.99	0.97	1.00	0.02
	San Miguel de Abona	0.93	0.84	0.97	0.05
	Santiago del Teide	3.03	2.98	3.08	0.07
	Vilaflor	0.57	0.49	0.73	0.14

**Table 4:** Mean concentrations, standard deviations (SD), maximum and minimum values of fluoride in water samples from Gran Canaria.

Area	Municipality	Mean Content (mg/L)	Min	Max
Metropolitan	Las Palmas	0.18 ± 0.13	0.04	0.38
South	Mogán	1.44 ± 1.33	0.03	2.99
	San Bartolomé de Tirajana	0.28 ± 0.78	0.03	3.51
Midlands	San Mateo	0.97 ± 0.06	0.90	1.03
	Valsequillo	1.44 ± 0.21	1.21	1.77
North	Teror	0.79 ± 0.23	0.42	1.18

**Table 5:** Estimated Daily Intake (EDI) and percentage contribution from consumption of Tenerife water supply by children (9–14 and 15–17 years old) and adults.

Region	Municipality	EDI (mg/Day)			Contribution _Children (15–17 Years) and Adults_ (%)			Contribution _Children (9–14 Years)_ (%)		
		1 L/Day	1.5 L/Day	2 L/Day	1 L/Day	1.5 L/Day	2 L/Day	1 L/Day	1.5 L/Day	2 L/Day
Metropolitan	El Rosario	1.62	2.44	3.25	23.2	34.8	46.4	32.5	48.7	65.0
	La Laguna	1.51	2.27	3.03	21.6	32.5	43.3	30.3	45.4	60.6
	Santa Cruz de Tenerife	0.73	1.10	1.46	10.4	15.7	20.9	14.6	21.9	29.2
	Tegueste	5.39	8.09	10.78	77.0	116	154	108	162	216
North	El Sauzal	7.00	10.51	14.01	100	150	200	140	210	280
	El Tanque	2.13	3.20	4.26	30.4	45.7	60.9	42.6	63.9	85.2
	Garachico	3.32	4.99	6.65	47.5	71.2	95.0	66.5	99.7	133
	Icod de los Vinos	2.52	3.78	5.04	36.0	54.0	72.0	50.4	75.6	101
	La Guancha	3.82	5.73	7.63	54.5	81.8	109	76.3	115	153
	La Matanza de Acentejo	2.85	4.27	5.69	40.7	61.0	81.3	56.9	85.4	114
	La Orotava	4.92	7.39	9.85	70.4	106	141	98.5	148	197
	La Victoria de Acentejo	2.71	4.06	5.42	38.7	58.1	77.4	54.2	81.3	108
	Los Realejos	1.63	2.44	3.25	23.2	34.9	46.5	32.5	48.8	65.1
	Los Silos	2.41	3.62	4.83	34.5	51.7	68.9	48.3	72.4	96.5
	Puerto de la Cruz	0.75	1.12	1.50	10.7	16.0	21.4	15.0	22.4	29.9
	San Juan de la Rambla	2.73	4.09	5.46	39.0	58.5	78.0	54.6	81.9	109
	Santa Úrsula	2.75	4.12	5.50	39.3	58.9	78.5	55.0	82.5	110
	Tacoronte	0.42	0.63	0.84	6.0	9.0	12.1	8.4	12.7	16.9
South	Adeje	0.27	0.41	0.55	3.9	5.9	7.8	5.5	8.2	10.9
	Arafo	2.32	3.49	4.65	33.2	49.8	66.4	46.5	69.7	93.0
	Arico	0.84	1.26	1.68	12.0	18.0	24.0	16.8	25.2	33.6
	Arona	0.11	0.17	0.22	1.6	2.4	3.2	2.2	3.3	4.5
	Candelaria	0.96	1.44	1.92	13.7	20.6	27.5	19.2	28.8	38.4
	Fasnia	2.60	3.90	5.20	37.1	55.7	74.2	52.0	77.9	104
	Granadilla de Abona	1.16	1.74	2.31	16.5	24.8	33.1	23.1	34.7	46.3
	Güimar	0.99	1.48	1.97	14.1	21.1	28.2	19.7	29.6	39.4
	San Miguel de Abona	0.93	1.40	1.86	13.3	20.0	26.6	18.6	27.9	37.2
	Santiago del Teide	3.03	4.55	6.06	43.3	65.0	86.6	60.6	90.9	121
	Vilaflor	0.57	0.86	1.15	8.2	12.3	16.4	11.5	17.2	22.9

**Table 6:** Estimated daily intake (EDI) and percentage contribution from consumption of Gran Canaria water supply by children (9–14 and 15–17 years old) and adults.

Region	Municipality	EDI (mg/Day)			Contribution _Children (15–17 Years) and Adults_ (%)			Contribution _Children (9–14 Years)_ (%)		
		1 L/Day	1.5 L/Day	2 L/Day	1 L/Day	1.5 L/Day	2 L/Day	1 L/Day	1.5 L/Day	2 L/Day
Metropolitan	Las Palmas	0.18	0.27	0.36	2.6	3.9	5.1	3.6	5.4	7.2
South	Mogán	1.44	2.16	2.88	20.6	30.9	41.1	28.8	43.2	57.6
	San Bartolomé de Tirajana	0.28	0.42	0.56	4.0	6.0	8.0	5.6	8.4	11.2
Medianías	San Mateo	0.97	1.46	1.94	13.9	21	28	19	29	39
	Valsequillo	1.44	2.16	2.88	20.6	31	41	29	43	58
North	Teror	0.79	1.19	1.58	11.3	17	23	16	24	32

## Data Availability

Data are contained within the article.
